# Identification of diagnostic signatures associated with immune infiltration in Alzheimer’s disease by integrating bioinformatic analysis and machine-learning strategies

**DOI:** 10.3389/fnagi.2022.919614

**Published:** 2022-07-29

**Authors:** Yu Tian, Yaoheng Lu, Yuze Cao, Chun Dang, Na Wang, Kuo Tian, Qiqi Luo, Erliang Guo, Shanshun Luo, Lihua Wang, Qian Li

**Affiliations:** ^1^Department of Neurology, The Second Affiliated Hospital of Harbin Medical University, Harbin, China; ^2^Department of Gerontology, The First Affiliated Hospital of Harbin Medical University, Harbin, China; ^3^Department of General Surgery, Chengdu Integrated Traditional Chinese Medicine and Western Medicine Hospital, Chengdu, China; ^4^Department of Neurology, Peking Union Medical College Hospital, Chinese Academy of Medical Sciences, Beijing, China; ^5^West China Medical Publishers, West China Hospital, Sichuan University, Chengdu, China

**Keywords:** Alzheimer’s disease, diagnostic biomarkers, immune cell infiltration, bioinformatic analysis, machine-learning strategies

## Abstract

**Objective:**

As a chronic neurodegenerative disorder, Alzheimer’s disease (AD) is the most common form of progressive dementia. The purpose of this study was to identify diagnostic signatures of AD and the effect of immune cell infiltration in this pathology.

**Methods:**

The expression profiles of GSE109887, GSE122063, GSE28146, and GSE1297 were downloaded from the Gene Expression Omnibus (GEO) database to obtain differentially expressed genes (DEGs) between AD and control brain samples. Functional enrichment analysis was performed to reveal AD-associated biological functions and key pathways. Besides, we applied the Least Absolute Shrinkage Selection Operator (LASSO) and support vector machine-recursive feature elimination (SVM-RFE) analysis to screen potential diagnostic feature genes in AD, which were further tested in AD brains of the validation cohort (GSE5281). The discriminatory ability was then assessed by the area under the receiver operating characteristic curves (AUC). Finally, the CIBERSORT algorithm and immune cell infiltration analysis were employed to assess the inflammatory state of AD.

**Results:**

A total of 49 DEGs were identified. The functional enrichment analysis revealed that leukocyte transendothelial migration, cytokine receptor interaction, and JAK-STAT signaling pathway were enriched in the AD group. MAF basic leucine zipper transcription factor F (MAFF), ADCYAP1, and ZFP36L1 were identified as the diagnostic biomarkers of AD with high discriminatory ability (AUC = 0.850) and validated in AD brains (AUC = 0.935). As indicated from the immune cell infiltration analysis, naive B cells, plasma cells, activated/resting NK cells, M0 macrophages, M1 macrophages, resting CD4^+^ T memory cells, resting mast cells, memory B cells, and resting/activated dendritic cells may participate in the development of AD. Additionally, all diagnostic signatures presented different degrees of correlation with different infiltrating immune cells.

**Conclusion:**

MAFF, ADCYAP1, and ZFP36L1 may become new candidate biomarkers of AD, which were closely related to the pathogenesis of AD. Moreover, the immune cells mentioned above may play crucial roles in disease occurrence and progression.

## Introduction

Alzheimer’s disease (AD) is the most well-known form of dementia, accounting for 50–60% of all cases, which is characterized by memory deficits, cognitive dysfunction, and behavioral impairment (Joe and Ringman, 2019). In 2018, Alzheimer’s Disease International revealed that the dementia prevalence is about 50 million people worldwide, projected to triple in 2050 (Scheltens et al., 2021). For decades, the conventional diagnosis for AD relies on deposits of amyloid beta (Aβ)-tau interaction and accumulation of neurofibrillary tangles in focal brain biopsy (Beach et al., 2012). Although the recent technological advancements in genetic testing and neuroimaging, opened doors to earlier diagnosis of AD (Basaia et al., 2019), there is still a lack of accurate and effective biomarkers for detection of AD prior to clinical onset.

Emerging studies suggest neuroinflammation is associated with AD and may play crucial role in AD progression in recent years, which involves not only the activation of brain resident immune cells but also the infiltration of peripheral activated immune cells (Heneka et al., 2015). A whitepaper workgroup has been established to delineate the dynamic response of peripheral–central immune crosstalk in AD (Bettcher et al., 2021). B cells, as primary effector cells, could infiltrate into the central nervous system (CNS) of AD and interact with CNS resident cells, participating in the immune response, central-peripheral signaling, and blood-brain barrier disruption (Bettcher et al., 2021). CD4^+^ T cells are the predominant type of T cells that are involved in AD progression, glial pro-inflammatory responses were reported to be driven by Th1 and Th17 and regulated by Th2 cells (McQuillan et al., 2010). Natural killer (NK) cells are known for their capability of killing tumors or virally infected cells, previous studies have indicated a role for NK cells in the pathogenesis of AD by mediating Aβ-dependent cytotoxicity (Solerte et al., 2000). Mast cells are implicated in neuroinflammation and neurodegenerative diseases by releasing several inflammatory mediators, cytokines, and chemokines (Kempuraj et al., 2017b). The levels of blood dendritic cells (DCs) are dysregulated and associated with increased severity of AD-related symptoms (Ciaramella et al., 2016). Thus, investigating immune cell infiltration associated with AD pathology is of great significance for AD treatment and may offer a novel insight forward in hopes of manipulating disease onset and progression. Furthermore, the association between AD diagnostic feature genes and immune cell infiltration is less understood.

In the present study, we obtained datasets of AD brains from the Gene Expression Omnibus (GEO) database and screened candidate diagnostic genes by machine learning methods, including the Least Absolute Shrinkage Selection Operator (LASSO) and support vector machine-recursive feature elimination (SVM-RFE) algorithms. We then evaluated and validated the expression levels and diagnostic abilities of these genes. Finally, the association between these feature genes and multiple kinds of infiltrating immune cells was explored, which may not only provide a new way to gain insights into the molecular mechanisms underlying the pathogenesis of AD, but also a perspective regarding the effective therapeutic targets of the disease.

## Materials and methods

### Dataset collection and preparation

We obtained and downloaded the five AD microarray expression profile datasets (GSE109887, GSE122063, GSE28146, GSE1297, and GSE5281) from the National Center for Biotechnology Information (NCBI) Gene Expression Omnibus (GEO) database,^1^ which is a public functional biomedical and genomic information repository. A search of mRNA profiles in AD was conducted with the following key words: (“Alzheimer’s disease” and “Expression profiling by array”), and the species was restricted as “Homo sapiens.” A total of 146 AD brain tissue and 93 healthy controls were merged into a training metadata cohort from four datasets (GSE109887, GSE122063, GSE28146, and GSE1297), profiled separately on the platform GPL10904 (Illumina HumanHT-12 V4.0 expression beadchip), GPL16699 (Agilent-039494 SurePrint G3 Human GE v2 8×60K Microarray 039381), GPL570 (Affymetrix Human Genome U133 Plus 2.0 Array), and GPL96 (Affymetrix Human Genome U133A Array). Subsequently, the probes in each dataset were annotated and transformed into gene symbols based on corresponding platform annotation files, and the probes without matching gene symbols were removed. For more than one probe corresponding to the same gene symbol, the average value was calculated as the final expression value. The R package “SVA” containing the “Combat” function (Johnson et al., 2007) was applied to compensate for the batch effect. In addition, the gene expression file of 87 AD brains and 74 control samples from the AD dataset GSE5281 was considered as the validation cohort for further analysis.

### Differential gene expression analysis

After removing batch effects using the “SVA” R package, we analyzed differentially expressed genes (DEGs) between 146 AD brain tissues and 93 healthy controls using the “limma” R package (Ritchie et al., 2015; Varma, 2020). The fold change (FC) >1.5 and adjusted *p* < 0.05 were regarded as thresholds for DEGs Screening.

### Gene set enrichment analysis

Gene set enrichment analysis (GSEA) (Subramanian et al., 2007) was performed to identify the most significant regulated Gene Ontology (GO) terms and Kyoto Encyclopedia of Genes and Genomes (KEGG) functional pathways between the AD and control groups. A gene set was regarded as significantly enriched if a *p* < 0.05 and false discovery rate <0.025.

### Machine learning for the diagnostic feature genes

We adopted two machine learning algorithms to perform the disease status predictions. A least absolute shrinkage and selection operator (LASSO)-based algorithm was utilized to identify the feature genes associated with the discrimination of AD and healthy controls using the “glmnet” R package (Friedman et al., 2010; Kang et al., 2021). To identify the set of genes with the highest discriminative power, support vector machine-recursive feature elimination (SVM-RFE) was applied using “e1071” R packages (Guo et al., 2014; Huang et al., 2014). The final candidate genes were obtained by the intersection of the genes from the two algorithms and the DEGs in the validation cohort. The expression levels of candidate genes were further tested in the validation cohort.

### Assessment of the diagnostic value of candidate biomarkers in Alzheimer’s disease

We tested the predictive value for diagnostic biomarkers using receiver operating curve (ROC) analysis, which was generated adopting expression data from 146 AD and 93 healthy brain tissues. To evaluate the diagnostic effectiveness, the area under the receiver operating characteristic curve (AUC) was utilized to distinguish AD from control samples and further verified in the validation cohort (Seshan et al., 2013).

### Evaluation of the relative fraction of immune cell subtypes

Infiltrating immune cells based on the gene expression profiles of AD data were calculated by using the CIBERSORT algorithm.^2^ A reference set with 22 sorted types of immune cell subtypes (LM22) with 1,000 permutations was used to reckon the relative abundance of infiltrating immune cells (Newman et al., 2015; Zhao et al., 2020). The discrepancy and correlation of infiltrating immune cells between the AD and control samples were analyzed and visualized by the R package “corrplot” and “vioplot.”

### Exploring the correlation between identified biomarkers and infiltrating immune cells

The association between the identified gene biomarkers expression and the levels of infiltrating immune cells was estimated by Spearman’s correlation analysis in R software and visualized using the chart technique with R package “ggplot2” (Wilkinson, 2011).

## Results

### Identification of differentially expressed genes in Alzheimer’s disease brain tissues

**FIGURE 1:**
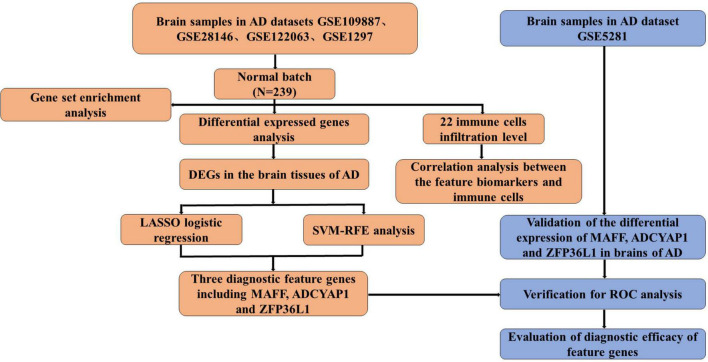
The flowchart of the analysis process.

**FIGURE 2:**
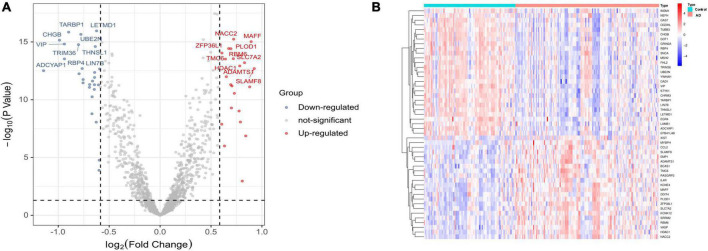
Differentially expressed genes (DEGs) identified between Alzheimer’s disease and control brain tissues. **(A)** Volcano plot. **(B)** Heatmap.

The flowchart of this study is shown in Figure 1. The expression profile data from four GEO datasets (GSE109887, GSE122063, GSE28146, and GSE1297) were composed of a total of 239 samples including 146 AD and 93 control brain samples. After we preprocessed and removed batch effects, a total of 49 DEGs were obtained, including 22 upregulated genes and 27 downregulated genes in the AD samples compared with the normal samples (Figure 2A), in which the remarkable difference was presented by heatmap (Figure 2B).

### Functional enrichment analysis of differentially expressed genes

**FIGURE 3:**
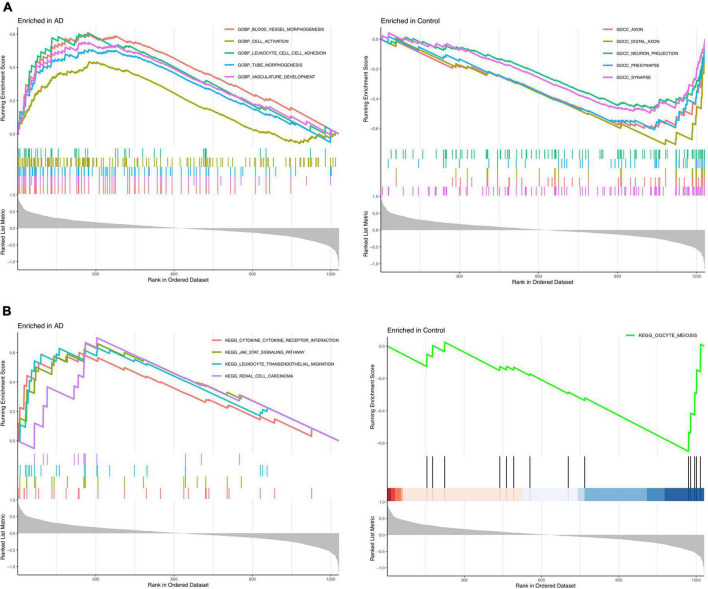
Enrichment analysis to investigate the potential function of differentially expressed genes (DEGs). **(A)** GSEA_GO analysis in Alzheimer’s disease (AD) or control group. **(B)** GSEA_KEGG analysis in AD or control group.

The GSEA_GO results revealed that blood leukocyte cell adhesion, cell activation, vessel morphogenesis, tube morphogenesis, and vasculature development were enriched in the AD group, while synapse, neuron projection, presynapse, axon, and distal axon were enriched in the healthy control group (Figure 3A). The GSEA_KEGG results revealed that leukocyte transendothelial migration, cytokine receptor interaction, JAK-STAT signaling pathway, and renal cell carcinoma were enriched in the AD group, while only oocyte meiosis was enriched in the healthy control group (Figure 3B). These findings strongly indicated that neuroinflammation and immune response play essential roles in the pathogenesis of AD.

### Identification of immune related diagnostic feature biomarkers in Alzheimer’s disease

**FIGURE 4:**
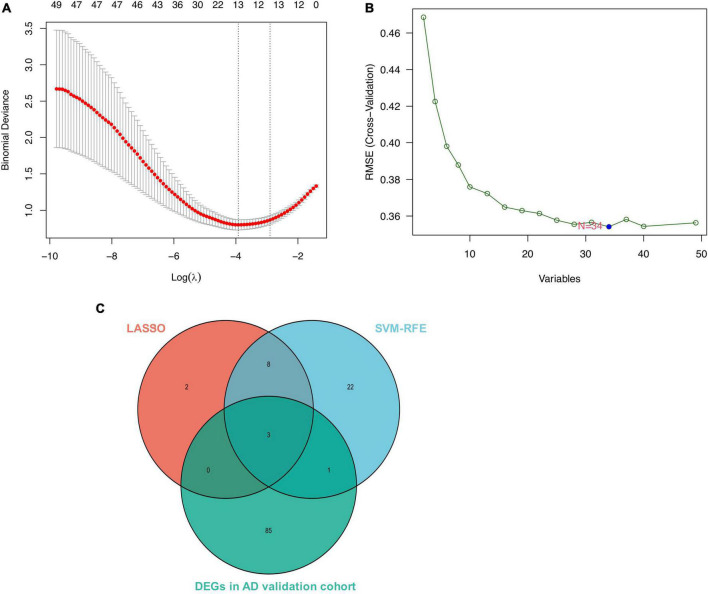
Screen for potential biomarkers of Alzheimer’s disease (AD) diagnosis. **(A)** Identified genes using Least Absolute Shrinkage Selection Operator (LASSO) algorithm. **(B)** The optimal feature biomarkers selection *via* support vector machine-recursive feature elimination (SVM-RFE) algorithm. **(C)** Venn diagram displaying one diagnostic marker intersected by LASSO, SVM-RFE algorithms, and differentially expressed genes (DEGs) in AD validated brain.

**FIGURE 5:**
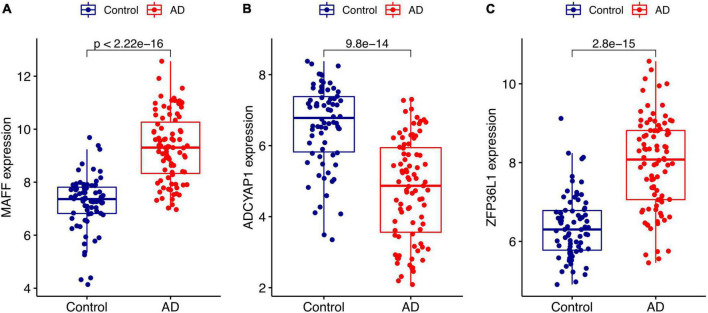
Validation of the expression levels of diagnostic biomarkers in the GSE5281 dataset. **(A)** MAFF expression level. **(B)** ADCYAP1 expression level. **(C)** ZFP36L1 expression level.

We conducted two different bioinformatic algorithms to screen the potential biomarkers of AD. By using the LASSO regression algorithm, DEGs were narrowed down to 13 variables as diagnostic biomarkers for AD (Figure 4A). By using the SVM-RFE algorithm, we identified a subset of 34 features among the DEGs (Figure 4B). The overlapping feature genes (MAF basic leucine zipper transcription factor F (MAFF), ADCYAP1, and ZFP36L1) among LASSO, SVM-RFE algorithm and DEGs in the validation cohort were ultimately selected for further study (Figure 4C). The expression levels of these three biomarkers (MAFF, ADCYAP1, and ZFP36L1) were further examined in the validation cohort GSE5281 to generate more accurate and reliable results, which were reported to be significantly dysregulated in AD compared with those in the control group. MAFF and ZFP36L1 showed significant upregulation, while ADCYAP1 showed significant downregulation in the AD group (*p* < 0.001; Figure 5).

### Diagnostic effectiveness of the feature biomarkers

**FIGURE 6:**
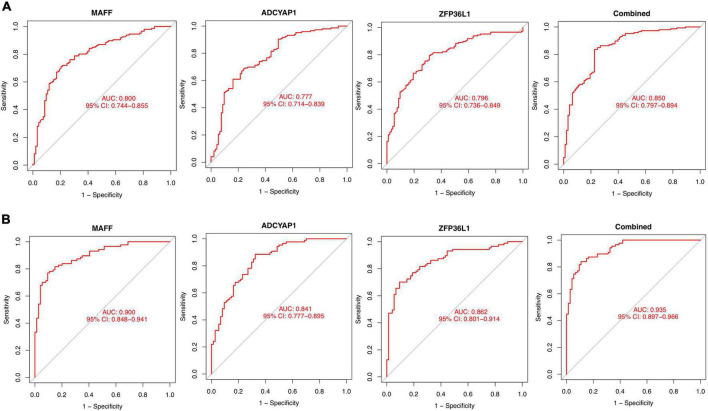
Diagnostic effectiveness of feature biomarkers. **(A)** Receiver operating curve (ROC) curves of candidate biomarkers (MAFF, ADCYAP1, ZFP36L1, and combined) in the training cohort. **(B)** ROC curves of candidate biomarkers (MAFF, ADCYAP1, ZFP36L1, and combined) in the validation cohort.

The identified genes were used to construct the diagnostic model using a logistic regression algorithm. We further quantified the discrimination ability by AUC. As shown in Figure 6A, the feature biomarkers demonstrated a high diagnostic power in discriminating AD brains from the control samples, with an AUC of 0.800 (95% CI 0.744–0.855) in MAFF, AUC of 0.777 (95% CI 0.714–0.839) in ADCYAP1, AUC of 0.796 (95% CI 0.736–0.849) in ZFP36L1, When the three genes were combined into one variable, the diagnostic ability in terms of AUC was 0.850 (95% CI 0.797–0.894) in the meta-data cohort. Moreover, the diagnostic efficiency of these biomarkers was confirmed in the validation cohort with an AUC of 0.900 (95% CI 0.848–0.941) in MAFF, AUC of 0.841 (95% CI 0.777–0.895) in ADCYAP1, AUC of 0.862 (95% CI 0.801–0.914) in ZFP36L1. Importantly, the diagnostic value of the three biomarkers combined yielded an AUC of 0.935 (95% CI 0.897–0.966; Figure 6B), further reinforcing the diagnostic ability of these three feature genes to serve as potential biomarkers in the discrimination of AD.

### Immune cell infiltration

**FIGURE 7:**
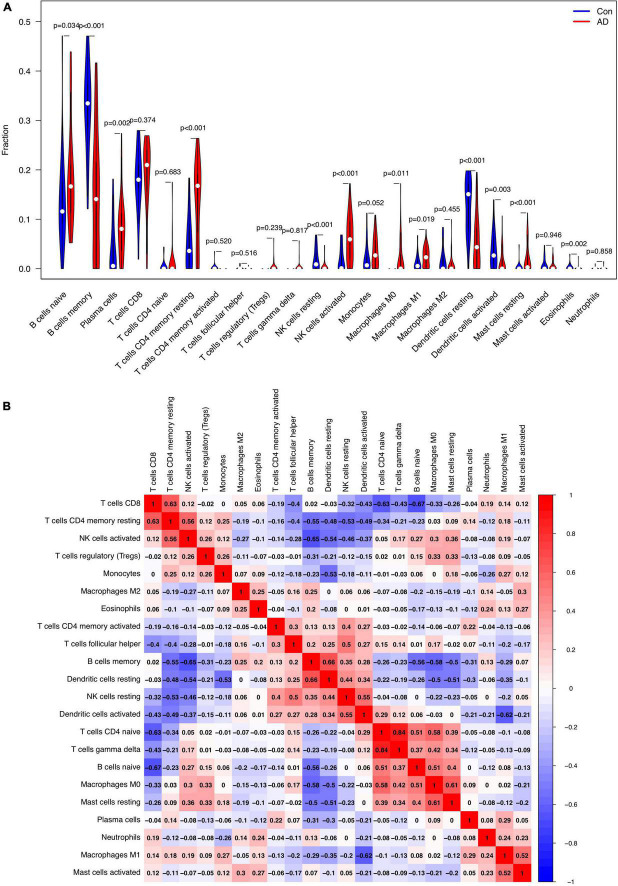
Comparison and correlation of immune cell infiltration. **(A)** Comparison of 22 infiltrated immune cell subtypes between Alzheimer’s disease (AD) and control brain tissues. Blue and red colors represent normal and AD samples, respectively. **(B)** Correlation analysis of these 22 immune cell subtypes mutually.

The relative proportion of 22 immune cells was estimated in each sample of AD cases and healthy controls using the CIBERSORT algorithm. In comparison with normal samples, AD samples generally contained a higher proportion of naive B cells (*p* = 0.034), plasma cells (*p* = 0.002), resting memory CD4+ T cells (*p* < 0.001), activated NK cells (*p* < 0.001), M0 macrophages (*p* = 0.011), M1 macrophages (*p* = 0.019), and resting Mast cells (*p* < 0.001), whereas the proportions of memory B cells (*p* < 0.001), resting NK cells (*p* < 0.001), resting DCs (*p* < 0.001), activated DCs (*p* = 0.003), and eosinophils (*p* = 0.002) were relatively lower (Figure 7A). As indicated from the correlation heatmap of the 22 types of immune cells, naive B cells and CD8+ T cells (*r* = −0.67, *p* < 0.001), activated NK cells and memory B cells (*r* = −0.65, *p* < 0.001), M1 macrophages and activated DCs (*r* = −0.62, *p* < 0.001) displayed the most significant negative correlations, respectively. Memory B cells and resting DCs (*r* = 0.66, *p* < 0.001), CD8+ T cells and resting memory CD4^+^ T cells (*r* = 0.63, *p* < 0.001), M0 macrophages and resting mast cells (*r* = 0.61, *p* = 0.002) displayed the most significant positive correlations, respectively (Figure 7B).

### Correlation analysis between the feature biomarkers and immune cells

**FIGURE 8:**
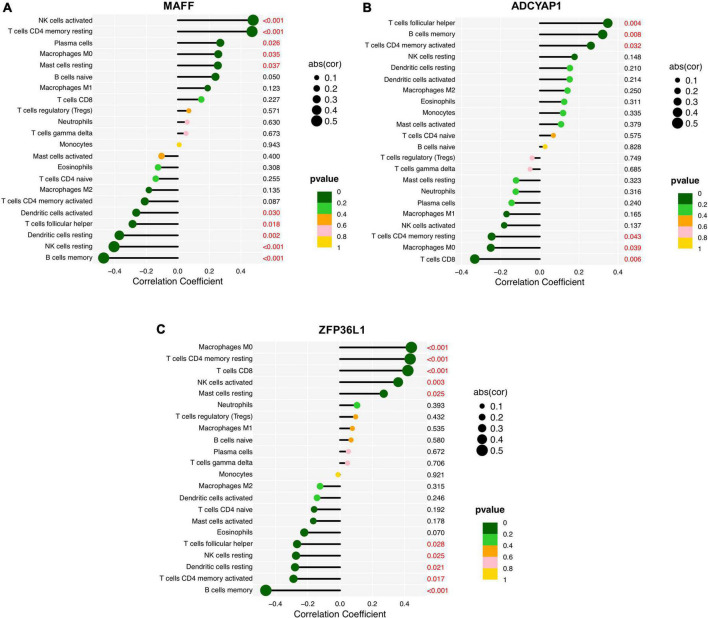
Correlation analysis between the feature biomarkers and infiltrating immune cells in Alzheimer’s disease (AD). **(A)** MAFF. **(B)** ADCYAP1. **(C)** ZFP36L1.

Finally, we assessed the relationship between the feature biomarkers and 22 types of immune cells using Spearman’s correlation analysis. Based on the results of correlation analysis, MAFF displayed a positive correlation with activated NK cells (*r* = 0.48, *p* < 0.001), resting memory CD4^+^ T cells (*r* = 0.47, *p* < 0.001), plasma cells (*r* = 0.27, *p* = 0.026), M0 macrophages (*r* = 0.26, *p* = 0.035), resting Mast cells (*r* = 0.25, *p* = 0.037) and showed a negative correlation with memory B cells (*r* = −0.47, *p* < 0.001), resting NK cells (*r* = −0.40, *p* < 0.001), resting DCs (*r* = −0.37, *p* = 0.002), follicular helper T cells (*r* = −0.29, *p* = 0.018), activated DCs (*r* = −0.26, *p* = 0.030) (Figure 8A). ADCYAP1 displayed a positive correlation with follicular helper T cells (*r* = 0.35, *p* = 0.004), memory B cells (*r* = 0.32, *P* = 0.008), activated memory CD4^+^ T cells (*r* = 0.26, *p* = 0.032) and showed a negative correlation with CD8^+^ T cells (*r* = −0.33, *p* = 0.006), M0 macrophages (*r* = −0.25, *p* = 0.039), resting memory CD4^+^ T cells (*r* = −0.25, *p* = 0.043) (Figure 8B). ZFP36L1 displayed a positive correlation with M0 macrophages (*r* = 0.44, *p* < 0.001), resting memory CD4^+^ T cells (*r* = 0.43, *p* < 0.001), CD8^+^ T cells (*r* = 0.42, *p* < 0.001), activated NK cells (*r* = 0.36, *p* = 0.003), and resting Mast cells (*r* = 0.27, *p* = 0.025) and showed a negative correlation with memory B cells (*r* = −0.46, *p* < 0.001), activated memory CD4^+^ T cells (*r* = −0.29, *p* = 0.017), resting DCs (*r* = −0.28, *p* = 0.021), resting NK cells (*r* = −0.27, *p* = 0.025), follicular helper T cells (*r* = −0.27, *p* = 0.028) (Figure 8C).

## Discussion

Identifying AD-related diagnostic features is of great clinical significance for the early detection and intervention of AD. Increased attention has been paid to the neuroinflammation mechanism involved in AD propagation. In the current study, we constructed an integrated bioinformatic analysis to identify the diagnostic biomarkers that are associated with immune cell infiltration in patients with AD.

The functional enrichment analysis revealed that leukocyte transendothelial migration, cytokine receptor interaction, and the JAK-STAT signaling pathway were enriched in the AD group (Figure 3). In the inflammation process during AD, the migration of circulating leukocytes from blood vessels into the CNS involves a sequence of adhesion and activation events, including capturing and rolling, activation induced by chemokines, and transmigration (Rossi et al., 2011). This result suggests that robust immune alterations, inflammatory response, and immune cells are involved in the pathological process of AD, thereby causing neuronal degeneration or death in AD progression, leading to cognitive dysfunction.

Based on LASSO and SVM-RFE algorithms, MAFF, ADCYAP1, and ZFP36L1 were identified as diagnostic biomarkers of AD with high sensitivity and specificity (Figures 4–6). As one of the small Maf proteins (sMafs), MAFF (MAF basic leucine zipper transcription factor F) predominantly localizes in the nucleus and acts as the basic region leucine zipper-type transcription factor (Motohashi et al., 2000). MaFF was demonstrated to be the most responsive to Aβ-induced oxidative stress in AD among sMafs and may potentiate the suppression of antioxidation (Wang et al., 2020). A meta-analysis of AD revealed that overexpression of MAFF repressed the transcription of NF-E2-related factors 2-dependent gene, leading to various cellular function disorders in AD, including immune and inflammatory responses, metabolism, and cognitive dysfunction (Wang et al., 2017). ADCYAP1 (Adenylate Cyclase-Activating Polypeptide 1) encodes pituitary adenylate cyclase-activating peptide (PACAP), which is a bioactive neuropeptide with pleiotropic effects, functions as a neurotrophic factor, neuromodulator, and neurotransmitter (Tamas et al., 2012). Our results showed that ADCYAP1 expression is reduced in AD, which is consistent with that suggesting PACAP is downregulated in three different mouse models or human samples of AD (Wu et al., 2006; Han et al., 2014). PACAP has been shown to protect neurons against the toxic effects of Aβ in several studies (Onoue et al., 2002; Gui et al., 2003). Furthermore, treatment with PACAP could attenuate 80% of the Aβ-induced reduction of cell viability through an increase in cAMP and a deactivation in caspase-3 (Onoue et al., 2002). ZFP36L1 (Zinc finger protein 36, C3H type-like 1) is one of several Zinc Finger Protein 36 family members, which could negatively regulate the post-transcriptional expression of targeted mRNAs through 3’ untranslated regions, including many inflammatory mediators and senescence-associated secretory phenotypes, suggesting ZFP36L1 was involved in the regulation of inflammation and senescence (Hyatt et al., 2014; Herranz et al., 2015; Wang et al., 2015), but the exact role of ZFP36L1 in AD pathogenesis remains unknown and requires further study to be fully clarified.

In addition to the neuronal compartment, increasing evidence suggests that AD pathogenesis also includes strong interactions with immune alterations in the brain, which begin early and persist throughout the disease (Jevtic et al., 2017). To more specifically evaluate the effects of the immune system in AD, we used CIBERSORT to assess the process of immune infiltration in AD brains. The results revealed higher proportions of naive B cells, plasma cells, activated NK cells, M0 macrophages, M1 macrophages, resting CD4+ memory cells, and resting mast cells in the AD group, along with lower proportions of memory B cells, resting NK cells, resting/activated DCs, and eosinophils (Figure 7A), suggesting these cells may be related to the occurrence and progression of AD. B cells and plasma cells were suggested to contribute to producing immunoglobulins that target amyloid beta which may thus interfere with plaque formation and disease progression (Söllvander et al., 2015; Kim et al., 2021). In a mouse model of AD, therapeutic depletion of B cells at the onset of the disease was shown to retard AD progression (Kim et al., 2021). CD4+ T cells may contribute to AD pathology by interacting with microglia, orchestrating immune mechanisms, and facilitating amyloid clearance, thus offering opportunities for therapeutic interventions (Mittal et al., 2019). NK cells may produce multiple inflammatory cytokines and chemokines, NK-derived IFN-γ and TNF-α were negatively correlated with the cognitive derangement in AD (Solerte et al., 2000). In transgenic AD mouse models, depletion of NK cells could enhance neurogenesis, reduce neuroinflammation, and improve cognitive function, indicating that targeting NK cells might unlock novel strategies to combat AD (Zhang et al., 2020). Previous research efforts have highlighted that peripheral macrophages may migrate across the vascular wall and “home” to the brain, which closely resemble brain-resident microglia (Gate et al., 2010). Mast cells could detect amyloid plaque formation and respond before microglia under brain stress conditions for they could release prestored mediators (Kempuraj et al., 2017a) and contribute to the degree of AD (Shaik-Dasthagirisaheb and Conti, 2016). DCs are professional antigen-presenting cells with the characteristic of being capable to respond to and produce neurotrophins, such as brain-derived neurotrophic factors (Ciaramella et al., 2013). Nevertheless, DC’s exact mechanism and DC-based therapies continue to be unclear and are under investigation.

Furthermore, we investigated the correlations of infiltrating immune cells and diagnostic signatures, MAFF displayed a significant and strong correlation with activated/resting NK cells, resting memory CD4+ T cells, and memory B cells (Figure 8A). ADCYAP1 was significantly associated with follicular helper T cells, memory B cells, activated memory CD4+ T cells, CD8+ T cells, M0 macrophages, and resting memory CD4+ T cells (Figure 8B). ZFP36L1 displayed significant correlations to infiltrations of immune cells such as M0 macrophages, resting memory CD4+ T cells, CD8+ T cells, activated NK cells, and memory B cells (Figure 8C). Of note, immunomodulatory actions of the ADCYAP1 gene encoding the neuropeptide PACAP have been reported to inhibit macrophage production and release of inflammatory mediators such as TNF-α and IFN-γ, and modulate the immune status *via* shifting the CD4+ T cells toward a Th2 phenotype (Delgado et al., 2003; Waschek, 2013). However, there is no information concerning these sophisticated interacting processes of genes and immune cells, in depth research is urgently required into the underlying molecular mechanisms and functional significance of immune cell infiltration in AD based on the mentioned assumption.

Although these immune-related diagnostic feature genes in AD were observed and identified in this comprehensive bioinformatics study, several limitations should be mentioned. (1) This study is second mining and investigation of the GEO database and the metadata was derived from different version platforms, the batch of effect may not be thoroughly removed from the large sample size based on the normal batch. (2) Despite the three feature biomarkers demonstrating a high diagnostic power in discriminating AD brains from control samples, there was a lack of attention to the correlation between these biomarkers and different subtypes of AD. (3) Our study provided preclinical observations or speculations of three novel candidate biomarkers and immune-related molecular mechanisms underlying AD, which need to be verified in further studies.

## Conclusion

In brief, this study demonstrated that MAFF, ZFP36L1, and ADCYAP1 refer to diagnostic markers of AD, which may play central roles in disease initiation and progression and are thus promising molecular targets for diagnosis and treatment. This study also reported that naive B cells, plasma cells, activated/resting NK cells, M0 macrophages, M1 macrophages, resting CD4^+^ T memory cells, resting mast cells, memory B cells, and resting/activated DCs are likely to critically impact the occurrence and progress of AD, which may serve as potential targets for future immunotherapy in patients with AD that warrant in-depth explorations.

## Data availability statement

The datasets presented in this study can be found in online repositories. The names of the repository/repositories and accession number(s) can be found in the article/supplementary material.

## Author contributions

YT and QLi designed the study, conducted the bioinformatics, and wrote the article. YT searched the datasets, performed the data management, and drafted the article. YL, CD, YC, and NW contributed to the statistical analysis and the software used in the work. KT, EG, and QLu interpreted the data. QLi, LW, and SL supervised the manuscript. All authors contributed to the article and approved the submitted version.

## Footnotes

1
http://www.ncbi.nlm.nih.gov/geo

2
https://cibersortx.stanford.edu/
